# Impaired Performance of the Q175 Mouse Model of Huntington’s Disease in the Touch Screen Paired Associates Learning Task

**DOI:** 10.3389/fnbeh.2018.00226

**Published:** 2018-10-02

**Authors:** Tuukka O. Piiponniemi, Teija Parkkari, Taneli Heikkinen, Jukka Puoliväli, Larry C. Park, Roger Cachope, Maksym V. Kopanitsa

**Affiliations:** ^1^Charles River Discovery Services, Kuopio, Finland; ^2^CHDI Management/CHDI Foundation, Los Angeles, CA, United States; ^3^UK Dementia Research Institute at Imperial College London, Division of Brain Sciences, Department of Medicine, Imperial College London, London, United Kingdom

**Keywords:** Huntington’s disease, visuospatial, touch screen, paired associates learning, reinforcement, progressive ratio, motivation, mouse

## Abstract

Cognitive disturbances often predate characteristic motor dysfunction in individuals with Huntington’s disease (HD) and place an increasing burden on the HD patients and caregivers with the progression of the disorder. Therefore, application of maximally translational cognitive tests to animal models of HD is imperative for the development of treatments that could alleviate cognitive decline in human patients. Here, we examined the performance of the Q175 mouse knock-in model of HD in the touch screen version of the paired associates learning (PAL) task. We found that 10–11-month-old heterozygous Q175 mice had severely attenuated learning curve in the PAL task, which was conceptually similar to previously documented impaired performance of individuals with HD in the PAL task of the Cambridge Neuropsychological Test Automated Battery (CANTAB). Besides high rate of errors in PAL task, Q175 mice exhibited considerably lower responding rate than age-matched wild-type (WT) animals. Our examination of effortful operant responding during fixed ratio (FR) and progressive ratio (PR) reinforcement schedules in a separate cohort of similar age confirmed slower and unselective performance of mutant animals, as observed during PAL task, but suggested that motivation to work for nutritional reward in the touch screen setting was similar in Q175 and WT mice. We also demonstrated that pronounced sensorimotor disturbances in Q175 mice can be detected at early touch screen testing stages, (e.g., during “Punish Incorrect” phase of operant pretraining), so we propose that shorter test routines may be utilised for more expedient studies of treatments aimed at the rescue of HD-related phenotype.

## Introduction

Huntington’s disease (HD) is a late-onset neurological condition characterised by progressive cognitive impairment and motor disturbances (McColgan and Tabrizi, 2018). The prevalence of HD is approximately 1 in 10,000 in individuals of Western European descent, whereas in Asian populations, the incidence is much lower (Baig et al., 2016). HD arises due to autosomal dominantly inherited expansion of CAG trinucleotide repeats in the huntingtin (*HTT*) gene on chromosome 4, which results in the production of mutant HTT protein with abnormally long polyglutamine track at the N-terminus (MacDonald et al., 1993). Aggregation of mutated HTT negatively impacts multiple cellular processes, including transcription, translation, proteostasis and mitochondrial function (Jimenez-Sanchez et al., 2017). Axonal transport and synaptic function deficits are prominent in neurones affected by HD with striatal medium spiny neurones being particularly sensitive (Bunner and Rebec, 2016). HD is usually diagnosed in middle age (35–45 years of age), when first motor symptoms begin to appear, followed by fatal outcome within 20 years. Despite a well-established genetic underpinning of HD, the currently approved treatments are all symptomatic and as such, do not modify the disease progression (Mrzljak and Munoz-Sanjuan, 2015; Wyant et al., 2017).

To understand the mechanisms of HD pathogenesis and to establish experimental platforms for drug discovery, multiple lines of genetically altered mice have been generated that can be classified into three groups: (a) mice expressing truncated human *HTT* fragments, e.g., R6 lines; (b) mice expressing full-length human *HTT* modified by the insertion of variable numbers of CAG repeats, e.g., YAC128 or BACHD lines; and (c) knock-in models, in which CAG repeats are inserted into the endogenous mouse *Htt* gene, e.g., Hdh^Q92^ line (Menalled and Chesselet, 2002; Chang et al., 2015). There are several reasons to use knock-in models of HD. First, the placement of abnormally expanded CAG repeats into the endogenous mouse *Htt* gene context avoids overexpression artefacts. Second, although the phenotype in knock-in mice takes longer to develop and is relatively mild, this circumstance may be advantageous for designing longitudinal experiments and is mechanistically reminiscent of the late HD onset in humans.

**Figure 1:**
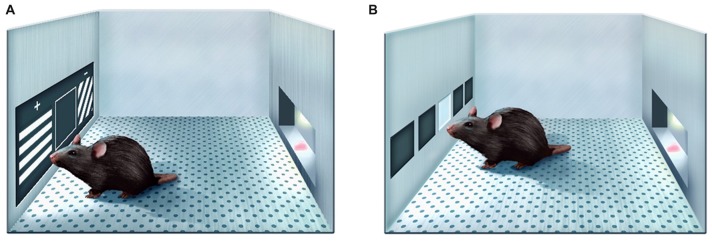
Schematic illustration of the paired associates learning (PAL) task **(A)** and Fixed/Progressive Ratio (FR/PR) task **(B)** in Bussey-Saksida touch screen operant chambers.

The knock-in Q175 model derives from Hdh^Q140^ line and has a spontaneous expansion of the CAG copy number in exon 1 of *Htt* (Menalled et al., 2012). Despite both heterozygous and homozygous Q175 mice generally have less aggressive phenotype than some other HD mouse models, they nonetheless recapitulate main manifestations of HD in humans, such as progressive accumulation of mutant huntingtin aggregates in striatal and cortical neurones, synapse loss, striatal and cortical atrophy, altered brain metabolic profile, decreased body weight and motor impairments (Oakeshott et al., 2011; Heikkinen et al., 2012; Menalled et al., 2012; Peng et al., 2016). Cognitive behaviour of Q175 mice was assessed by using two-choice swimming test, T-maze, and simple instrumental tasks that used lever presses and nosepokes to obtain nutritional reinforcement (Oakeshott et al., 2011, 2013; Heikkinen et al., 2012; Menalled et al., 2012; Whittaker et al., 2017). To make the results of behavioural evaluations of mouse models of HD more relevant to the clinical setting, it would be advantageous to apply testing techniques that have greater similarity to cognitive examinations of humans. Touch screen-based approach has a high translational value as unlike some more forceful techniques to assess cognitive functions in rodents, it is based on interactions with a touch-sensitive screen prompted by visual stimuli and nutritional rewards for correct responses (Bussey et al., 2012; Hvoslef-Eide et al., 2016). Q175 mice, as well as other HD mouse models, such as R6/2 and BACHD, were recently tested in touch screen chambers and found to exhibit age-dependent deficits in the acquisition of pairwise visual discrimination skills and in the reversal of visual discrimination learning (Morton et al., 2006; Farrar et al., 2014; Skillings et al., 2014; Curtin et al., 2015; Glynn et al., 2016). From the instrumental point of view, the touch screen-based setting is analogous to the one used for clinical assessment of cognitive impairment in humans, e.g., by the Cambridge Neuropsychological Test Automated Battery (CANTAB; Nithianantharajah and Grant, 2013). Many studies have utilised the paired associates learning (PAL) CANTAB task to evaluate cognitive functions in individuals affected by neurodegenerative diseases. In that task, during encoding phase, the participant has to memorise the locations on the screen of initially one and gradually up to eight unique patterns and then, during the retrieval, touch the correct white boxes, where each stimulus, now presented in the centre of the screen, was shown during encoding (Barnett et al., 2016). PAL task performance was found to be impaired in HD patients (Lange et al., 1995; Lawrence et al., 1996; Begeti et al., 2016). In the rodent version of the task (Talpos et al., 2009; Horner et al., 2013), animals demonstrate learning of the similar object-location relationship by selectively touching one (correct) out of two simultaneously presented images on the basis of its location on the screen (Figure 1A). Common neural basis of performance in rodents and humans has been inferred from lesion, pharmacological and genetic studies in the former and brain imaging studies in the latter (Barnett et al., 2016). This circumstance facilitates the translation of PAL results obtained in preclinical models to the clinical setting as it indicates similarity of behavioural processing strategies in humans and rodents. In the present study, we examined whether Q175 mice had deficits in the mouse version of PAL task. In addition, because performance of Q175 mice during PAL routine suggested altered motivation to perform food-rewarded touch screen tasks, we also compared the rates of sustained repetitive responding of Q175 and litter-matched wild-type (WT) mice in fixed ratio (FR) and progressive ratio (PR) operant tasks recently implemented in the touch screen chamber setting (Figure 1B; Heath et al., 2015, 2016).

## Materials and Methods

### Animals

All animal experiments were performed as specified in the licence authorised by the National Animal Experiment Board of Finland (Eläinkoelautakunta, ELLA) and according to the National Institutes of Health (Bethesda, MD, USA) guidelines for the care and use of laboratory animals.

Given that homozygosity for the extended CAG repeat is extremely rare in humans, we sought to assess cognitive phenotypes in mice heterozygous for the mutant knock-in allele. As it has been shown that cognitive performance in heterozygous Q175 mice progressively decreases with age (Curtin et al., 2015; Southwell et al., 2016), to maximise the chance to reveal phenotypes in touch screen tests, we chose to work with 10–11-month-old animals.

In the PAL experiment, the selected cohort comprised 14 male 10-month old Q175 mice heterozygous for the mutant *Htt*^tm1Mfc^ allele harbouring 185–210 CAG repeats (Menalled et al., 2012) and 15 age-matched male WT mice. We will henceforth refer to this subset of mutants by their original name, zQ175.

In the course of testing, one mutant mouse failed to complete the last pretraining stage and another mutant mouse acquired PAL very slowly, so it had to be excluded from the final PAL analysis. Thus, PAL performance was analysed for 15 WT and 12 zQ175 mice.

For the FR/PR test, we used a separate cohort of 12 male 11-month-old Q175FΔ*neo* mice (Southwell et al., 2016) and 11 WT littermates that were housed at 2–3 animals per cage. These animals had the floxed *neo* cassette upstream of exon 1 removed, whereas zQ175 mice used in the PAL test retained it^1^. We used this subtype of Q175 mice because during the allocated slot for FR/PR tests, only Q175FΔ*neo* mice were available that had age and sex comparable to those in the above described zQ175 cohort from the PAL test. Moreover, it has been shown that the phenotype of Q175FΔ*neo* mice is similar to that of the mutants retaining the *neo* cassette, although the changes become manifested at a slightly earlier age (Southwell et al., 2016; Heikkinen et al., 2017). In the end of the experiments, tail samples were sent to Laragen Inc. for genotype and CAG repeat number confirmation.

Animals were kept in a temperature- and humidity-controlled environment under a 13:11 h light/dark cycle (lights on at 07:00 am and off at 8:00 pm) at 22 ± 1°C. Cages (IVC type II, Allentown Inc., Allentown, NJ, USA) were kept at negative pressure and furnished with corn cob-derived bedding (The Andersons, Maumee, OH, USA), nesting material (aspen wool, Tapvei Oy, Kortteinen, Finland), a tinted polycarbonate tunnel (Datesand, Manchester, UK) and a petite green gumabone (BioServ, Flemington, NJ, USA). During the experiment, mice were kept on a restricted diet (Purina Lab Diet 5001) at 85%–90% of their free-feeding weight in order to maintain motivation for the task, with water *ad libitum*.

For the PAL test, mice received one 60-min long training session per day, whereas in the FR/PR test, animals were tested for up to 120 min daily. For both tests, testing proceeded in the afternoon hours, starting between 4:30 pm and 6:30 pm, 5–7 days per week.

### Food Deprivation

Before the start of the experiments, animals were gently handled and weighed. Access to food was gradually restricted, so that each animal was within 85%–90% of their free feeding weight. In addition, a small quantity of Valio Profeel strawberry-flavoured milk drink (Valio, Helsinki, Finland) was provided initially into the cages to accustom the animals with the flavour and taste of the reward to be used during testing. To maintain the weight of mice in the 85%–90% range throughout testing period, the animals received a rationed amount (typically 2.5–3.5 g) of standard lab pellets daily immediately after testing.

### Equipment

Touchscreen testing was performed in 24 Bussey-Saksida mouse touch screen operant chambers (Campden Instruments, Loughborough, UK) essentially as described (Horner et al., 2013; Heath et al., 2016). For the PAL and FR/PR tasks, the 3- and 5-window masks were used in front of the touch sensitive screen, respectively (Campden Instruments).

### Activity Assessment

On the first day of testing, after a 3-day gradual food deprivation period, the naïve mice were placed individually into Campden Instruments Ltd. touchscreen chambers for 30 min, with no rewards available. The total numbers of beam breaks (combined front and rear), traversals (number of times the mouse “traversed” the chamber, defined as a rear beam break followed by a front beam break), screen touches and nose pokes into the food magazine in 30 min were recorded.

### Pre-training for the PAL Task

Prior to the PAL test, the mice were trained on basic touchscreen task requirements, which were introduced gradually, as described previously (Horner et al., 2013, 2018). Following activity assessment, mice were first tested in a Pavlovian task (“Initial Touch” stage) that introduced several aspects of the touchscreen testing, including the relationship between visual stimulus presentation and reward availability. One out of 40 variously shaped images was presented randomly in one of the three response windows for 30 s. When the image disappeared, the magazine light turned on and a drop reward was delivered with a tone. If the mouse touched the image, it disappeared immediately and the mouse was rewarded with a larger reward portion concomitantly with a tone and magazine illumination. After a 20-s inter-trial interval (ITI), the next trial commenced. This stage was considered complete when 30 trials were completed in 1 h and all rewards were consumed.

At the next stage of pre-training (“Must Touch” stage), a visual stimulus was presented randomly in one of the three locations on the screen, and remained there until touched, introducing the requirement for mice to touch (nose-poke) the image on the screen. Doing so was rewarded (milkshake, tone, magazine light on). A 20-s ITI (magazine inactive, no image presented) occurred after the collection of the reward pellet, after which a new trial began with a presentation of the next image. This stage was considered complete when 30 trials are completed in 1 h.

Thereafter, the requirement to initiate trials was introduced (“Must Initiate” stage); sessions progressed as in the previous phase, but after the ITI, the magazine light was turned on, and mice had to nose poke into it to start the next trial. Successful initiation (here and in all subsequent task phases) was indicated by the extinction of the magazine light and appearance of an image on the screen. Again, this stage was considered complete when the mouse finished 30 trials in 1 h.

Finally, a “punishment” was introduced for touching the empty (plain black) location instead of the image providing a cue that signalled incorrect responses (“Punish Incorrect” stage). Sessions progressed as in the previous stage, except that if a mouse touched the empty location, it was “punished” with a 5 s “time out” (image disappeared, house light switched on, no reward). Following this, a 5 s correction ITI started after which mice could initiate a correction trial, to begin a correction procedure. In the correction procedure, the trial was repeated with the same stimuli in the same location until the mouse made the correct response. A correct response was rewarded in the usual way, the correction procedure ended, and after a standard 20 s ITI, mice were able to initiate a normal trial. After reaching a performance criterion of at least 75% of the 36 trials in a session correct (not including correction trials) and with 36 trials completed in under 60 min in two consecutive sessions, mice were moved on to the actual PAL task.

### PAL Task

During the PAL task, each daily 36-trial (or maximum 60-min long) session commenced with the requirement to initiate, as during pretraining. Doing so triggered presentation of a pair of images, one in two of the three windows (left, middle or right). The third window remained blank and non-responsive. There were three possible visual stimuli (“Lines Grid-Right,” “Lines Grid-Left” and “Vertical lines”) with dark and light lines going in different directions (Figure 1A). On each trial, the correct (S+) stimulus was determined by a combination of stimulus shape (the “object”) and its location, e.g., “Vertical lines” image was correct in the left location, “Grid-Right” image—in the middle, and “Grid-left image”—on the right. On each trial, one image was presented in its correct location along with one of the two alternative images in its incorrect location (S−), giving a total of 6 possible trial types. Visual stimuli remained on the screen until S+ or S− was touched, and were removed immediately following a touch to either. Touches to the blank inactive location were ignored. Response to S+ was rewarded (tone, reward drop of milkshake delivered, magazine light on, no “time out”); response to the S− was “punished” (house light on for a 5 s “time out,” no milkshake delivery). Incorrect responses to S− were followed by a correction procedure as described above. The task ITI was normally 20 s, but only 5 s prior to correction trials. No trial type was presented more than three times consecutively.

Mice were tested for 50 compound sessions, and their performance was analysed in blocks of five sessions (36 × 5 = 180 trials in total). The minimal possible duration of PAL testing was 53 days, because the first and second of the 50 sessions were deliberately split into 3 and 2 days (3 × 12 trials and 2 × 18 trials), respectively, to introduce the animals gradually to the PAL task. It should be noted that the precise definition of the PAL “session” in this study was the total time needed for the mouse to complete 36 trials: because many animals failed to complete the usually required 36 daily trials (Horner et al., 2018) for multiple days, particularly during the early stages of the PAL task, additional training days were given as required to ensure all mice were presented with exactly 1,800 trials in total.

Performance score (number of correct responses out of 36 trials in each session) was converted to a percentage correct score for each mouse, and these were averaged across 180 trials (i.e., blocks of five sessions). The numbers of correction touches, touches to the blank area, as well as response times and reward collection latencies were also recorded and analysed across blocks of five sessions.

### FR Training

Following activity assessment and completion of “Initial Touch” pre-training, identical to that used for mice in the PAL group, the animals in the *FR/PR* group underwent FR training during which animals learned to nose-poke the image initially once and then several times in a row to receive the reward (Heath et al., 2015, 2016). Animals were permitted a maximum of 60 min to complete 30 trials of FR training schedule. A single trial consisted of the presentation of a 4 × 4-cm white square stimulus in the central screen response location indefinitely (Figure 1B). Animals were required to touch the stimulus, which was then removed from the screen. A single reward was then delivered coincident with magazine illumination and tone delivery (1 s, 3 kHz). Animals were required to collect the reward from the magazine before the next trial would commence after a 4.5-s ITI. As one operant response was required to elicit a single reward, this schedule is referred to as FR1. To move to the next FR stage, animals had to complete 30 trials in a single session and consume all earned rewards.

Animals that fulfilled FR1 performance criterion were advanced to FR2 training. The FR2 schedule required producing two operant screen responses to earn a single reward. Repeated responding was reinforced by brief (500-ms) removal of the stimulus following successful screen contact and delivery of a distinct “chirp” tone (10 ms, 3 kHz). As with FR1 training, the criterion for advancement to the next stage required animals to complete 30 trials in a single session and consume all earned rewards.

Upon completion of FR2 performance criteria, animals were advanced to FR3 training that required emission of three operant screen responses to earn a single reward. Similarly, the criterion for advancement to the next, FR5 stage required animals to complete 30 trials in a single session and consume all earned rewards.

During FR5 stage, in addition to the requirement to complete 30 trials in a single session, mice are usually expected to demonstrate specificity of interaction with the target screen location over the other four never illuminated locations. For example, a target:blank touch ratio of at least 3:1 is recommended, which can be quickly achieved in young C57Bl/6J mice (Heath et al., 2015, 2016). However, in our pilot experiments, we found that some Q175Δ*neo* mice have difficulty achieving that level of specificity even after 15 daily sessions, whereas many WT mice attained it quicker. Therefore, to avoid large differences in the number of days spent on FR5 training between genotypes, we adopted more relaxed criteria for FR5 stage completion before advancing animals to PR testing: (i) all animals received at least five FR5 daily sessions; (ii) additional FR5 sessions were given after 5 days if the mice did not complete all 30 trials on three last consecutive days; and (iii) the total number of FR5 sessions was capped at 10.

Throughout FR training schedules, mice were left in the touchscreen chambers for 45–60 min, even if they completed the required number of trials within a shorter period. This was done to accustom animals to spending longer times in the chamber, as would be required during the subsequent PR testing. To assess performance during the last FR5 session in detail, we analysed schedule length, target and blank touch rates, target/blank touch ratio, post-reinforcement pause (time between head exit from food magazine after reward collection and the first touch on the next ratio), inter-touch interval, reward collection latency, as well as screen (front) and magazine (rear) infrared beam break rates.

### PR Testing

PR test is an effort-based task that allows determining in quantitative terms the motivation of the animal to expend physical effort to receive reinforcing stimulus, typically of nutritional nature (Markou et al., 2013). During PR schedule, the requirement to perform a certain number of elementary physical acts, e.g., lever presses in the original PR task (Hodos, 1961) or nose pokes to a touch-sensitive screen (Heath et al., 2015), gradually increases during the session. As a result, when the required effort becomes too high, cost/benefit calculations prompt the animal to cease responding, and the number of responses following the last rewarded response ratio, known as “breakpoint,” is used as a measure of perseveration.

In our experiments, animals were permitted a maximum of 120 min per session to complete as many trials as possible. The first trial of all PR sessions required a single operant screen response after which a single reward was delivered coincident with magazine illumination and tone delivery (1 s, 3 kHz). Animals were required to collect the reward from the magazine before the next trial commenced after a 4.5 s ITI. The response requirement was increased in all subsequent trials according to a linear ramp of 8 (1, 9, 17, 25…*n* + 8; PR8) with repeated touches supported by brief 500-ms removal of the screen stimulus following successful screen contact and delivery of a “chirp” tone (10 ms, 3 kHz). PR8 schedule ended if animals failed to make a screen touch or visit food magazine following reward delivery for 5 min or after 120 min, whichever was sooner.

Testing on PR8 schedule proceeded for seven consecutive days. In addition to breakpoint, the classical measure of PR task performance, we also analysed schedule length, target and blank touch rates, target/blank touch ratio, post-reinforcement pause, inter-touch interval, reward collection latency and front and rear infrared beam break rates.

### Statistical Analysis

Pairwise comparisons in groups with normally distributed values were done by the Student’s independent samples *t*-test, applying Welch’s correction for groups with unequal variances if necessary. In groups for which the assumption of normality was rejected by the D’Agostino-Pearson test, the non-parametric Mann-Whitney U-test was used for pairwise comparisons. One-sample *t*-test was used to assess the difference of the group mean from a theoretical value. In our previous study (Piiponniemi et al., 2017) we noticed that within-session reaction times and reward collection latencies were right-skewed even after log10 or square root transformations. Therefore, for between-genotype comparisons, session median rather than session mean values of these parameters from individual mice were used, because they were more robust to the effect of outliers and more representative as central tendency measures for each session. Datasets of repeated measurements were analysed by the two-way analysis of variance (ANOVA; within-subject factor—day/session; between-subject factor—genotype). In the case of statistically significant genotype × session interactions, *post hoc* Holm-Šidák multiple comparisons test was used to determine at which sessions (or blocks thereof) the difference between genotypes was significant. All statistical analyses were conducted with a significance level of 0.05 by using GraphPad Prism 7 (GraphPad Software Inc., La Jolla, CA, USA). Throughout the text, data are presented as the mean ± standard deviation.

## Results

### PAL Experiment

At the start of testing, zQ175 and WT mice in the PAL cohort were 306 ± 7.0 and 300.6 ± 4.9 days old, so the mutants were slightly but significantly older (*P* = 0.0456, Mann-Whitney test). Before the start of food restriction, the free-feeding mutant animals were lighter than WT animals (28.7 ± 2.09 g vs. 32.7 ± 1.65 g, respectively; *P* < 0.0001, Student’s *t*-test). The latter observation was in accordance with previously reported weight loss in zQ175 mice (Heikkinen et al., 2012).

#### Activity in PAL Cohort

**Figure 2:**
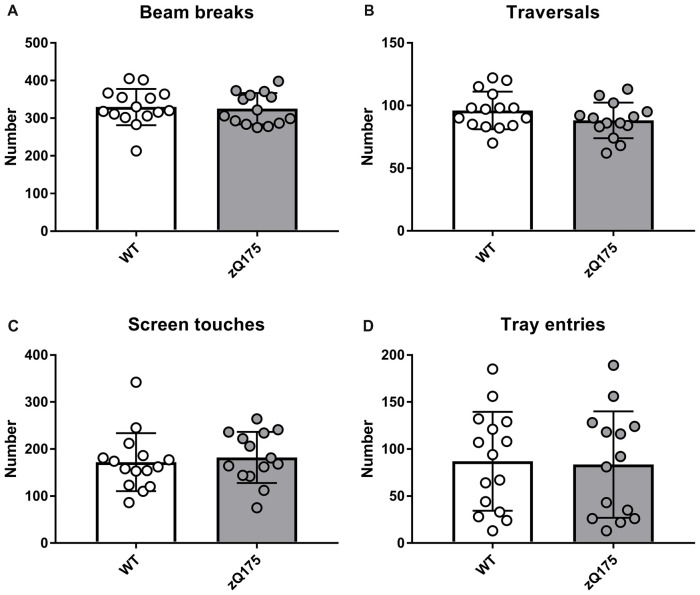
Locomotor activity of zQ175 and wild-type (WT) mice of the PAL cohort. Numbers of infrared beam breaks **(A)** chamber traversals **(B)** screen touches **(C)** and food magazine entries **(D)** made by animals of the two genotypes were not statistically different (*P* > 0.05; Student’s *t*-test or Mann-Whitney U-test, as appropriate). Data are presented as the mean ± standard deviation. Here and in other Figures, circles represent individual mouse data. N_zQ175_ = 14; N_WT_ = 15.

Overall locomotor activity of zQ175 mice did not differ from that of WT mice as they made a similar number of beam breaks and traversals during their first 30-min exposure to touchscreen chamber (Figures 2A,B). In addition, zQ175 and WT mice made similar numbers of screen touches and entries to the food tray (Figures 2C,D). Therefore, we concluded that at the age of 10 months, zQ175 mice did not exhibit major locomotor and anxiety phenotypes, which could nonspecifically affect their learning of the touch screen routine.

#### Pretraining for PAL Touch Screen Task

**Figure 3:**
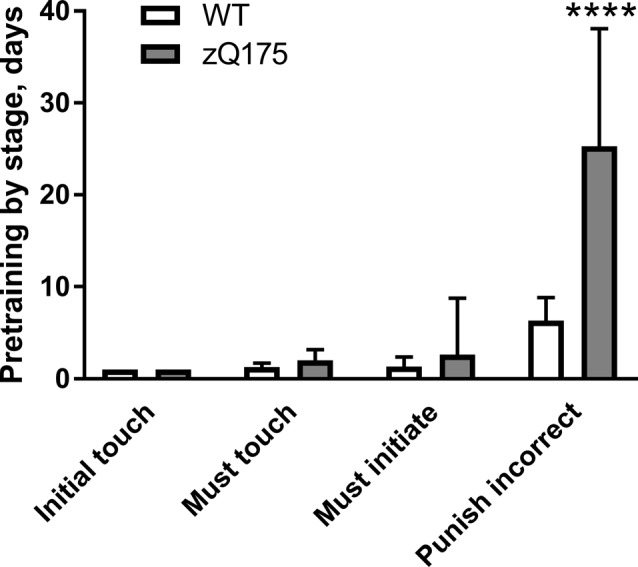
Duration of touch screen pretraining stages in zQ175 and WT mice (PAL cohort). Data are presented as the mean ± standard deviation. For the zQ175 mouse that failed to achieve Punish incorrect criterion within 50 daily sessions, the number of days at that stage was capped at 50. Statistical significance of differences is indicated as follows: ****P < 0.0001 (Student’s *t*-test with Welch’s correction). N_zQ175_ = 14; N_WT_ = 15.

Sequential introduction of basic operant learning steps during pretraining for PAL touch screen task showed that zQ175 mice performed comparably to WT counterparts during “Initial touch,” “Must touch” and “Must initiate” pretraining stages (*P* > 0.05 in all cases; Figure 3). However during the “Punish incorrect” stage, zQ175 mice required fourfold larger number of days than WT animals to achieve the criterion: 25.3 ± 12.6 vs. 6.3 ± 2.5 days, respectively (*P* < 0.0001, Student’s *t*-test with Welch’s correction; Figure 3). Moreover, one zQ175 mouse failed to achieve the criterion for “Punish incorrect” stage even after 50 days, whereupon it was excluded from further testing.

**Figure 4:**
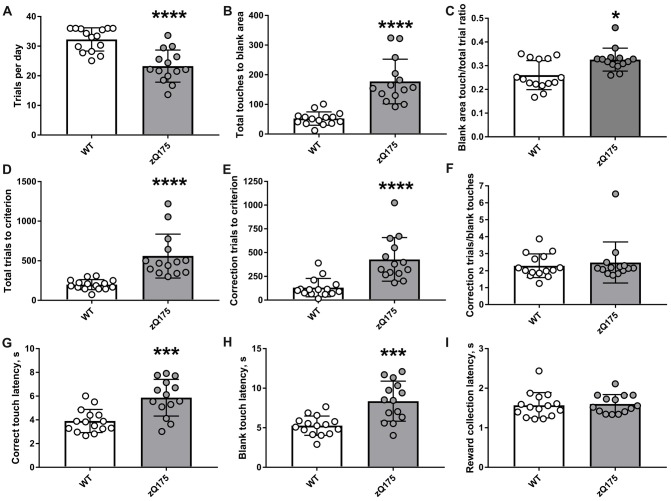
Detailed analysis of performance during Punish incorrect stage of touch screen pretraining in zQ175 and WT mice. **(A)** Average number of Punish incorrect trials per day. **(B)** Total number of touches to blank area made until reaching the stage criterion. **(C)** The ratio of touches to blank area to the total number of trials. **(D)** Total number of trials to criterion. **(E)** Total number of correction trials to criterion. **(F)** The ratio of correction trials to the total number of touches to blank area. **(G)** Median latency to do correct (image) touch. **(H)** Median latency to do blank area touch. **(I)** Median latency to collect the reward from food magazine. Data are presented as the mean ± standard deviation. Statistical significance of differences is indicated as follows: *P < 0.05; ***P < 0.001; ****P < 0.0001 (Student’s *t*-test, Student’s *t*-test with Welch’s correction, or Mann-Whitney test, as appropriate). N_zQ175_ = 14; N_WT_ = 15.

Protracted attainment of the “Punish incorrect” stage criterion by zQ175 mice stemmed from two principal causes (Figure 4). First, during “Punish incorrect” stage of pretraining, zQ175 mice more frequently failed to complete the required number of trials per day. Second, mutant animals poked into the image with insufficient selectivity, making an unacceptably high number of touches to the blank parts of the screen while an image was present in one of the three windows). In particular, we found that although each animal was given a chance to complete 36 trials per day during pretraining, zQ175 mice completed on average only 23.3 ± 5.4 “Punish incorrect” trials daily (Figure 4A), which was a significantly lower trial rate than that of WT animals (32.3 ± 3.9; *P* < 0.0001, Student’s *t*-test). Furthermore, zQ175 mice made significantly more touches to blank area than WT animals (Figure 4B). Because mutant mice had to complete more trials due to relatively lower operant responding, we also compared blank area touch rates, i.e., the ratio of blank area touches to total trials and found that zQ175 mice exhibited a higher relative blank touch rate than did WT animals (Figure 4C). The combination of lower daily trial rate and reduced accuracy of responding led to a significantly higher number of total trials (image touch trials + blank touch trials) by zQ175 mice to complete “Punish incorrect” stage (Figure 4D). In addition, zQ175 mice received more correction trials, i.e., when following a blank area touch, the trial was repeated with the same stimuli in the same location until the mouse made the touch to the image (Figure 4E). The difference in correction trials could be explained by the higher number of initial blank area touches as well as by repeated poking into unilluminated parts of the screen during several correction trials in a row. We found that zQ175 mice unlikely had a strong preference for unilluminated parts of the screen (or, in other words, aversion to the lit part of the screen), because the ratio of total correction trials to total number of blank touches in mutant mice was comparable to that in WT animals (Figure 4F).

We also analysed latencies of several behavioural reactions in zQ175 and WT mice during the “Punish incorrect” stage and found that mutants were slower to poke both into the image (correct response, Figure 4G) and into the blank area of the screen (incorrect response, Figure 4H) after the image had appeared. At the same time, zQ175 and WT mice collected reward with similar latencies (Figure 4I).

#### PAL Task Performance

**Figure 5:**
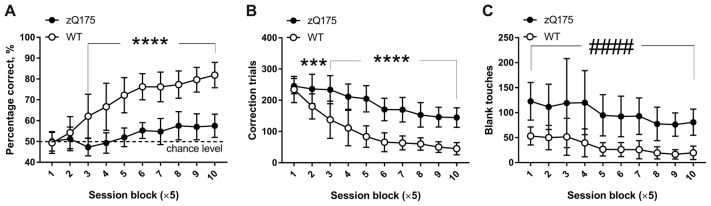
Main parameters of PAL task performance in zQ175 and WT mice. **(A)** Percentage of correct responses during 50 consecutive PAL sessions pooled across 10 blocks of five sessions each. In some cases, especially during early stages of PAL task, the 36 session trials had to be given across several days (see “Materials and Methods” section for detailed explanation). **(B)** Total number of correction trials across 10 blocks of five PAL sessions each. **(C)** Total number of touches to blank area across 10 blocks of five PAL sessions each. Data are presented as the mean ± standard deviation. Data were analysed by the two-way ANOVA (within-subject factor—session block; between-subject factor— genotype). In the case of statistically significant genotype × session interactions, *post hoc* Holm-Šidák multiple comparisons test was used to determine at which blocks of sessions the difference between genotypes was significant (***P < 0.001; ****P < 0.0001). If genotype × session interaction was not significant, main genotype effect was indicated as follows: ^####^*P* < 0.0001. N_zQ175_ = 12; N_WT_ = 15.

During pretraining for the PAL task, mice learned to poke into the window displaying a random image and to suppress poking into the remaining blank, non-illuminated two windows. Therefore, when the animals were progressed to the actual PAL task (Figure 1A), their initial performance fluctuated around 50% correct (chance) level because they poked randomly into one of the two simultaneously displayed images, only one of which was in the correct location. In the course of the 50 PAL sessions, WT mice demonstrated clear improvement of their object-location associative learning from chance level in the beginning of testing to over 80% correct response rate during the last five sessions (Figure 5A). In contrast, zQ175 mice showed deficient acquisition of the PAL task, as their performance, even at later stages, was only slightly higher than chance level (10th session block: 58.0 ± 5.6%, *P* = 0.0007, one-sample *t*-test against theoretical mean of 50%) and much lower than the percentage of correct responses of WT animals (Figure 5A). There was a significant interaction between the effects of genotype and session on the percentage of correct response (*F*_(9,225)_ = 20.36, *P* < 0.0001, mixed model repeated measures ANOVA) with the values being significantly different between genotypes at session blocks 3–10 (Figure 5A; *P* < 0.0001, *post hoc* Holm-Šidák multiple comparisons test). Furthermore, zQ175 mice received more correction trials following touches to “S−” (Figure 5B). As during the “Punish incorrect” pretraining stage, mutant mice touched blank window more frequently than did WT mice (Figure 5C; main effect of genotype *F*_(1,25)_ = 69.7; *P* < 0.0001, mixed model repeated measures ANOVA).

**Figure 6:**
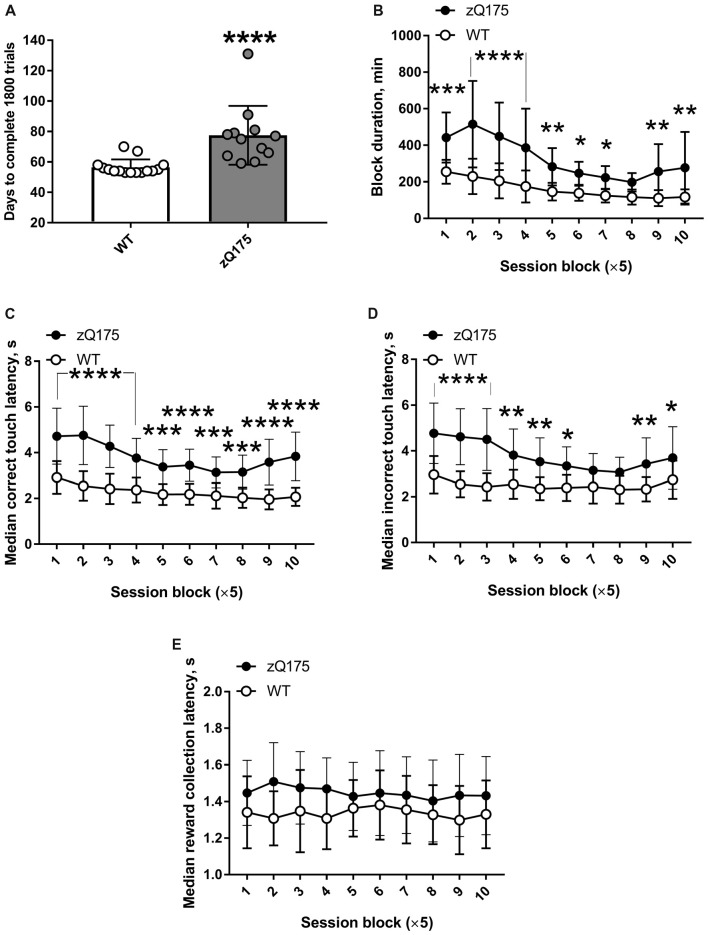
Decelerated performance of PAL task by zQ175 mice. **(A)** Number of days required to complete the 1,800 PAL trials (50 sessions of 36 trials each). **(B)** Duration of the block of five sessions at different stages of PAL task. **(C)** Median latency to touch the correct image. **(D)** Median latency to touch the incorrect image. **(E)** Median latency to collect the reward from food magazine. Data are presented as the mean ± standard deviation. Statistical significance of differences is indicated as follows: *P < 0.05; **P < 0.01; ***P < 0.001; ****P < 0.0001 (Mann-Whitney test **(A)** or *post hoc* Holm-Šidák multiple comparisons test after significant genotype × session interactions were revealed by two-way repeated measures ANOVA **(B–D)**). N_zQ175_s = 12; N_WT_ = 15.

zQ175 mice required significantly longer time to complete the PAL task than did WT mice (Figure 6A). The shortest possible duration of PAL testing was 53 days, because the first and second of the 50 sessions were deliberately split into 3 and 2 days (3 × 12 trials and 2 × 18 trials), respectively, to introduce the more demanding requirement to associate images with a particular location more gradually. Whereas only two out of 15 WT mice required more than 60 days to complete 50 sessions (36 trials each), only two out of 14 zQ175 mice completed it within 60 days (Figure 6A). One zQ175 animal required more than 3 days on average to complete a session, i.e., it was doing less than 12 trials per day. Experiments with that mouse were stopped when it had completed about half of the required sessions, and these data were not used in PAL analysis. zQ175 mice had significantly longer PAL sessions at all experimental stages, except for session block 8 (genotype × session block interaction: *F*_(9,225)_ = 5.557, *P* < 0.0001; Figure 6B). Mutant animals displayed consistently longer latencies to touch the correct visual stimulus (genotype × session block interaction: *F*_(9,225)_ = 3.364, *P* = 0.0007; Figure 6C). Similarly, the latencies of the first touch to the incorrect image in a pair were also longer in zQ175 mice during all sessions except for session blocks 7 and 8 (genotype × session block interaction: *F*_(9,225)_ = 3.764, *P* = 0.0002; Figure 6D). At the same time, neither genotype (*F*_(1,25)_ = 3.086, *P* = 0.0912) nor session block (*F*_(9,25)_ = 0.695, *P* = 0.714) significantly affected the latency to collect the reward (Figure 6E). The latter finding suggested that longer image touch latencies of zQ175 mice were task-specific deficits and not a consequence of some generalised motor impairment.

### FR/PR Experiment

The slower rate of responding and frequent failure to complete the required moderate number of trials during pretraining and during actual PAL task in zQ175 mice could reflect deficient reinforcement learning and/or lower motivation. The latter construct can be more selectively (i.e., without the confound of the learning deficit) assessed in effort-based tasks, using, for example, FR and PR schedules (Hodos, 1961; Markou et al., 2013). Therefore, using a separate cohort of 11-month-old related Q175Δ*neo* mice and their WT littermates, we examined their performance in the recently developed touch screen version of FR and PR tasks (Heath et al., 2015, 2016).

At the start of this experiment, litter-matched Q175Δ*neo* and WT mice in the FR/PR cohort were 329 days old on average. Before the start of food restriction, the free-feeding weight of Q175Δ*neo* mice in this cohort was lighter than that of WT littermates (29.0 ± 1.4 g vs. 34.0 ± 2.0 g, respectively; *P* < 0.0001, Student’s *t*-test). This phenotype has been also observed in the original article about Q175FΔ*neo* mice (Southwell et al., 2016). As in the case of the PAL cohort (Figure 2), no significant differences were found between Q175Δ*neo* and WT mice in any of the activity parameters measured during the first exposure to touch screen chamber (*P* > 0.05 for all four measures, data not shown).

#### FR Test Performance

**Figure 7:**
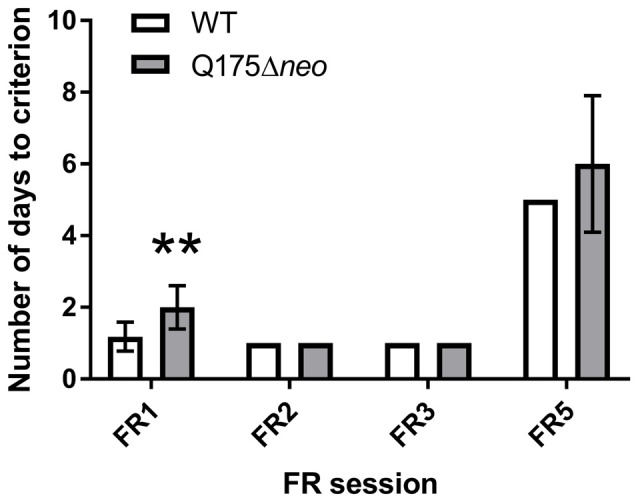
Performance of Q175Δ*neo* and WT mice during FR schedules. Data are presented as the mean ± standard deviation. Q175 dataset includes data from two mutant mice that failed to consistently complete 30 FR5 trials and the number of their FR5 sessions was capped at 10. Statistical significance of differences is indicated as follows: **P < 0.01 (Mann-Whitney test). N_Q175_Δ*neo* = 12; N_WT_ = 11.

**Figure 8:**
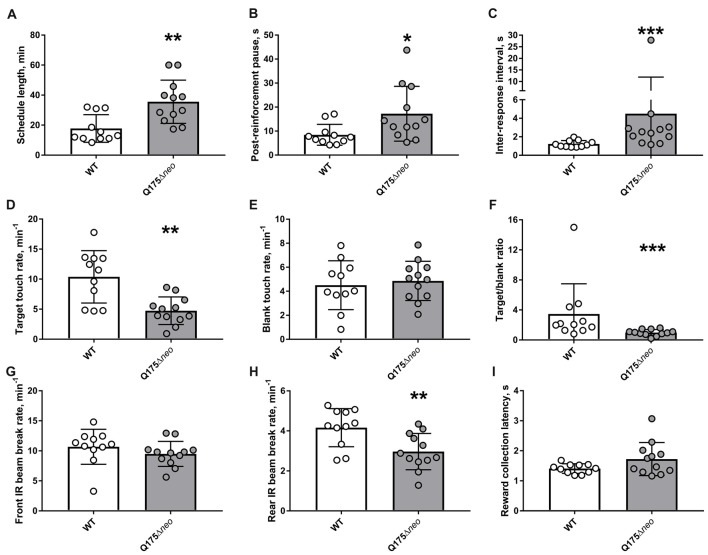
Detailed analysis of performance during the last FR5 session. **(A)** Last FR5 session length. **(B)** Median post-reinforcement pause. **(C)** Median inter-response interval. Y-axis was split for better illustration of all individual values. **(D)** Target touch rate. **(E)** Blank touch rate. **(F)** The ratio of the number of target touches to the number of touches to blank area. **(G)** Front infrared (IR) beam break rate. **(H)** Rear IR beam break rate. **(I)** Median latency to collect the reward from food magazine. Data are presented as the mean ± standard deviation. Statistical significance of differences is indicated as follows: *P < 0.05; **P < 0.01; ***P < 0.001; Student’s *t*-test, Student’s *t*-test with Welch’s correction, or Mann-Whitney test, as appropriate. N_Q175_Δ*neo* = 12; N_WT_ = 11.

The majority of WT mice achieved FR1 criterion in 1 day, whereas most Q175Δ*neo* mice required at least 2 days for that (Figure 7). All mice, irrespective of the genotype, completed FR2 and FR3 task criteria within 1 day. Mutant animals required nominally more days to achieve FR5 criterion. However, the difference did not achieve statistical significance (*P* = 0.093, Mann-Whitney U-test). Partly, it was because we capped the maximum number of FR5 sessions to 10: 2 out of 12 Q175Δ*neo* mice still failed to complete all 30 trials during 60 min of FR5 testing on FR5 days 8–10. There were numerous differences in the dynamics and specificity of FR schedule responding between Q175Δ*neo* and WT mice and to illustrate them, we analysed the performance of mice during the last FR5 session, whereupon the animals were moved to PR testing. Last FR5 session length was significantly longer in mutant animals (Figure 8A). The protracted performance was due to longer post-reinforcement pause (the period between head exit from the food magazine following reward collection and the first touch in the next trial) and slower responding during the trial evidenced by longer inter-response intervals (times between the images being touched; Figures 8B,C).

Target touch rate was significantly lower in Q175Δ*neo* mice, whereas blank touch rate was similar between genotypes (Figures 8D,E). However, as the session length was considerably longer in Q175Δ*neo* mice, they made significantly more blank touches over the course of the whole session (161 ± 62 vs. 78 ± 49, *P* = 0.001, Mann-Whitney U-test). Thus, target/blank touch ratio for the whole session was significantly lower in Q175Δ*neo* mice (Figure 8F). These data suggested that Q175Δ*neo* mice exhibited lower specificity of the interaction with the relevant part of the touch screen, making relatively fewer target touches per given time.

Whereas the activity near the screen during the last FR5 session was similar between genotypes, rear IR beam break rate was relatively slower in Q175Δ*neo* mice (Figures 8G,H). It is unlikely that the latter difference was associated with decreased motivation to work for reward: mutant and WT animals collected the reward with very similar latencies (Figure 8I). Moreover, the rates of empty magazine entries were similar in Q175Δ*neo* mice and in WT littermates (0.88 min^−1^ vs. 1.00 min^−1^, *P* = 0.45, Mann-Whitney U-test), indicating that mutants actively sought the nutritional reward.

To verify if the above described differences were not considerably distorted by the data from the two Q175Δ*neo* mice that failed to complete all 30 trials during their last three FR5 sessions, we also carried out the same comparisons having excluded those two animals. All the statistically significant genotype effects with the exception of the longer post-reinforcement pause in Q175 mice remained significant (data not shown), so we advanced all mutant animals to the PR schedule.

#### PR Test Performance

**Figure 9:**
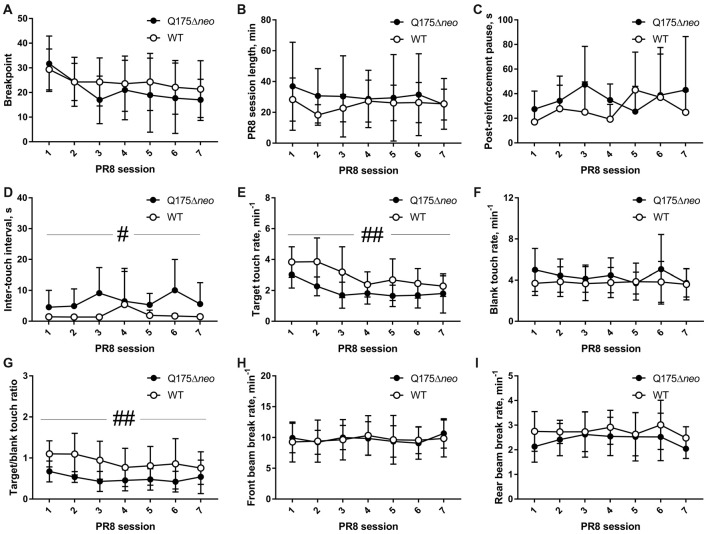
Performance of Q175Δ*neo* and WT mice during consecutive daily PR sessions on PR8 schedule. **(A)** Breakpoints. **(B)** Lengths of daily sessions. **(C)** Median post-reinforcement pause values. **(D)** Median inter-response intervals. **(E)** Target touch rates. **(F)** Blank touch rates. **(G)** Target/blank touch ratio values. **(H)** Front infrared (IR) beam break rates. **(I)** Rear IR beam break rates. Data are presented as the mean ± standard deviation (in **(C,D)** due to large variability, only the upper part of standard deviation range is provided for clarity). Genotype × session interactions were not significant. Main genotype effect is indicated as follows: ^#^*P* < 0.05; ^##^*P* < 0.01. For **(A,B, E–I)** N_Q175_Δ*neo* = 12; N_WT_ = 11. For analyses in **(C,D)** N_Q175_Δ*neo* = 8; N_WT_ = 10, as data from mice that completed less than two ratios in any of the seven PR schedule days were excluded.

Both Q175Δ*neo* and WT mice vigorously emitted operant responses during PR8 sessions. Whereas there was a significant effect of session on breakpoint value (*F*_(6,126)_ = 5.054, *P* = 0.0001), genotype did not affect breakpoints (Figure 9A). Genotype, session number or interaction of these factors did not significantly affect the length of daily sessions that were stopped after 5 min of inactivity in most cases (Figure 9B). One Q175Δ*neo* mouse kept responding for 2 h on the first day of PR8 schedule, so its session length was capped at this value for analysis. Post-reinforcement pauses before the start of new ratios were similar in both genotypes (Figure 9C), but median inter-touch intervals were slightly but significantly longer in Q175Δ*neo* mice (*P* = 0.046, main genotype effect, Figure 9D). As was the case during FR5 sessions, target touch rate was slower in mutant animals (genotype effect: *F*_(1,21)_ = 11.19, *P* = 0.0031; session effect: *F*_(6,126)_ = 10.46, *P* < 0.0001; Figure 9E), whereas neither genotype nor session affected blank touch rates (Figure 9F). Target/blank touch ratio was gradually decreasing in the course of testing on PR8 schedule (session effect: *F*_(6,126)_ = 3.681, *P* = 0.0021) and overall lower in Q175Δ*neo* mice (genotype effect: *F*_(1,21)_ = 10.84, *P* = 0.0035; Figure 9G). Neither front beam nor rear beam rates were affected by genotype or PR8 session (Figures 9H,I). We also analysed directional behaviour toward reward magazine and found no differences in reward collection latency (genotype effect: *F*_(1, 20)_ = 2.109, *P* = 0.162; session effect: *F*_(6,120)_ = 1.06, *P* = 0.39; data from one Q175Δ*neo* mouse were excluded as on 1 day, it did not collect the reward). Furthermore, neither genotype nor session significantly affected the rate of empty food magazine visits (genotype effect: *F*_(1,21)_ = 2.099, *P* = 0.1621; session effect: *F*_(6,126)_ = 1.847, *P* = 0.095).

## Discussion

In the present study, we show a pronounced deficit in object location/PAL in 10–11-month-old heterozygous zQ175 mice, an animal model of HD. Besides high rate of errors, we also noted considerably lower responding rate in mutants during the last stage of pretraining and during the PAL task itself. Our examination of effortful operant responding during FR and PR reinforcement schedules in closely related Q175Δ*neo* mice of similar age confirmed slower and unselective performance of mutant animals observed during the PAL task but suggested that motivation to work for nutritional reward in Q175Δ*neo* and WT mice was similar.

Visuospatial deficits have been extensively documented in HD patients (Mohr et al., 1991; Lawrence et al., 2000; Dumas et al., 2013; Pirogovsky et al., 2015; Corey-Bloom et al., 2016). These findings have been conceptually recapitulated in animal models of HD. Deficient spatial learning in Morris water maze has been noted in R6/1 (Brooks et al., 2012), R6/2 (Lione et al., 1999), YAC128 (Brooks et al., 2012a), HdhQ92 (Brooks et al., 2012c), HdhQ111 (Giralt et al., 2012), HdhQ150 (Brooks et al., 2012b) HD mouse models and in transgenic HD rats (Kirch et al., 2013), whereas individuals with HD perform poorly in the human versions of the water maze (Majerová et al., 2012; Begeti et al., 2016). Learning of delayed matching to position and delayed non-matching to position tasks was deficient in HdhQ111 and zQ175 mice (Curtin et al., 2015; Yhnell et al., 2016b) as well as in transgenic HD monkey (Chan et al., 2014), mirroring deficits in similar tests in symptomatic HD patients (Lange et al., 1995; Lawrence et al., 2000).

However, differences in preclinical animal and clinical test settings often preclude straightforward comparisons of the changes observed in rodents and individuals with HD. For example, learning in Morris water maze is driven by the negative reinforcement (such as stress), which may adversely affect the performance and is incompatible with assessing cognition in humans. Application of touch screen technology for testing cognition in rodents and other species facilitates such cross-species evaluations: touch screen tests have been used for diagnosing cognitive dysfunction in humans for decades, although it should be remembered that they were partly inspired by the desire to utilise cognitive testing routines originally developed in rodents and monkeys (Barnett et al., 2016). Recent refinement of touch screen-based techniques enabled successful replication of human cognitive phenotypes in mice with homologous mutations and even permitted reverse, mouse-to-human, translation (Nithianantharajah et al., 2013, 2015).

Here, for the first time, we applied touch screen PAL task to a mouse model of HD. Impaired performance of zQ175 mice in this task (Figure 5) has important translational parallels, as disruptions of verbal and pattern-location associative learning in HD patients have been demonstrated in various tests, including CANTAB (Lange et al., 1995; Sprengelmeyer et al., 1995; Rich et al., 1997; Lawrence et al., 2000; Begeti et al., 2016). In human CANTAB PAL task, the performance of individuals is usually assessed by the number of trials required to correctly locate all patterns, the number of errors made and the memory score, representing the overall number of patterns correctly located after the first presentation (Lawrence et al., 2000). Compared to healthy control subjects, HD patients use more trials, commit more errors and demonstrate lower memory score, typically experiencing considerable difficulty at the six- and eight-pattern stages of the task (Lange et al., 1995; Lawrence et al., 2000; Begeti et al., 2016). These deficits are directly comparable to the significantly lower number of correct responses and higher number of correction trials observed in zQ175 mice compared to those in WT counterparts (Figure 5). Persistently low percentage of correct responses of zQ175 mice in the course of PAL task was likely caused by disturbances in multiple cognitive domains. As discussed by Lawrence et al. (2000), visuospatial tasks require several distinct processes: (1) attendance to and discrimination between the stimuli; (2) acquisition of the learning rule, including the realisation that correct responses are associated with reward, and memory of the rule throughout testing; (3) matching the available set of stimuli to the sample in memory; and (4) appropriate response selection. Furthermore, successful performance in positively reinforced visuospatial tasks ultimately depends on inherent motivation to work for corresponding reward.

Impaired ability to discriminate between two visual images in touch screen chambers had been noted in 26-week old but, surprisingly, not in 48-week old zQ175 mice (Farrar et al., 2014; Curtin et al., 2015). In R6/2 mice, age- and CAG repeat number-dependent impairments in pairwise visual discrimination have been shown (Morton et al., 2006; Glynn et al., 2016). These mouse phenotypes are reminiscent of compromised perception of visual stimuli in HD patients (Büttner et al., 1994; Jacobs et al., 1995; O’Donnell et al., 2008). However, as argued by Lawrence et al. (2000), it is unlikely that HD patients have a generalised perceptual deficit as their poor performance in perceptual tests, e.g., in simultaneous matching-to-sample, might be due to attentional deficits.

In our experiments, longer latencies to touch correct and incorrect images in PAL task in zQ175 mice (Figures 6C,D) as well as longer post-reinforcement pause in FR5 task in Q175Δ*neo* mice (Figure 8B) suggest that decreased attention may also contribute to impaired acquisition of PAL. Deficits in rapid attention to visual stimuli in other knock-in HD mouse models were also demonstrated by using 5-choice serial reaction time task (Trueman et al., 2009, 2012; Yhnell et al., 2016a,c). These data are in accord with reduced attentional capacity of HD patients (Finke et al., 2007; Georgiou-Karistianis et al., 2012; Hart et al., 2015).

Notably, post-reinforcement pauses were not significantly different between WT and Q175Δ*neo* mice during testing on PR8 schedule (Figure 9C), which could reflect eventual recovery of this deficit upon continuous training. Positive effects of training on the discriminatory ability and attention of Q175 and R6/2 mice have been reported previously (Curtin et al., 2015; Yhnell et al., 2016c).

Poor rule learning likely was also a factor in deficient performance of zQ175 mice in PAL task. Pronounced deficits in operant responding of mutants were already evident at the last “Punish incorrect” stage of pretraining for PAL task (Figures 3, 4): mutant mice required many more trials to learn to selectively touch the image, while withholding interactions with blank windows. This deficit may be explained by impaired proactive selective stopping, a process that involves activation of striatal, pallidal and frontal areas in humans, which is disturbed in HD patients (Majid et al., 2013). Similar deficit has not been reported during instrumental pretraining of zQ175 and R6/2 HD mice for the pairwise discrimination touch screen task, likely because the “Punish incorrect” step was not used (Morton et al., 2006; Farrar et al., 2014; Curtin et al., 2015). Acquisition of the simple nose poke response (akin to “Must Touch” pretraining stage used here) was impaired slightly in 53-week and 74-week old heterozygous zQ175 mice and nearly completely abrogated in homozygous mutants (Oakeshott et al., 2011). Furthermore, zQ175, R6/2 and BAC HD mice exhibited deficits in a simple visual Go/No-Go task that required animals to nose-poke into a recess for food reward in the presence of light, but to withhold responding in the absence of the reinforcer (Oakeshott et al., 2013). In addition, impaired operant rule learning in delayed alternation task has been reported in HdhQ92 mouse model of HD (Trueman et al., 2009). These findings are in accord with numerous reports on deficient rule learning in individuals with HD (Knopman and Nissen, 1991; Lange et al., 1995; Filoteo et al., 2001).

Our data on the performance of Q175Δ*neo* mice in FR and PR tasks were somewhat unexpected. On the one hand, protracted execution of FR task (Figures 8A,B) and longer inter-touch intervals during both FR and PR schedules (Figures 8C, 9D) were in line with decelerated performance of similar mutants in the PAL task (Figure 6). Psychomotor slowing in zQ175 mice was also noted in touch screen visual discrimination task (Farrar et al., 2014), so these data collectively demonstrate that Q175 mouse lines can be used for modelling HD-related bradykinesia (Thompson et al., 1988; Sánchez-Pernaute et al., 2000). On the other hand, indices related to the motivation, such as breakpoint, the rate of empty food magazine visits, or reward collection latency, were similar between genotypes (Figures 8I, 9A), despite an earlier study of zQ175 mice, which utilised a lever-equipped apparatus, revealed lower breakpoints in 30-week old mutants (Covey et al., 2016). In a similar setting, 27–33-month-old zQ175 mice showed slower response rates and reduced number of earned reinforcements on PR schedules (Oakeshott et al., 2012; Curtin et al., 2015). In the latter studies, in the absence of clear breakpoints, those measures were deemed to reflect lower motivation. Lower response (target touch) rate in Q175Δ*neo* mice was noted also in our experiments (Figures 8D, 9E), however, the breakpoints, which correlate with the number of earned rewards, were similar in WT and mutant animals (Figure 9A). Lower breakpoint values were also described in HdhQ111 mice (Yhnell et al., 2016a; Minnig et al., 2018).

The discrepancies between breakpoint data in this study and published reports likely stem from a differential setting (touch screen chambers vs. lever-equipped operant boxes or operant buckets) and steeper, PR8, reinforcement schedule than those employed in other studies in which breakpoints were achieved (Covey et al., 2016; Yhnell et al., 2016a; Minnig et al., 2018). Also, there was a subtle genetic difference between Q175Δ*neo* mice in our experiments and zQ175 mice used in published studies. Generally, mice emitted relatively fewer responses in the touch screen version of PR task (compare breakpoints in Figure 9A per 120 min of testing with Figure 1A of Covey et al. (2016)), which implies that touch screen testing was more strenuous. However, it is then unclear why this circumstance did not make detection of phenotype in Q175Δ*neo* mice easier. It must be noted though that the strategies to achieve similar breakpoint values in WT and Q175Δ*neo* mice were different, as mutants made target touches relatively less frequently. Nonetheless, our results suggest that modelling apathetic behaviour in Q175 mouse lines by testing them on PR schedules in touch screen chambers is less optimal than using lever-equipped chambers.

Despite similar breakpoints were observed during testing on PR8 schedule in a related Q175 line (Figure 9A), motivational deficits may still have affected the performance of zQ175 mice during the PAL task. Because mutants made more errors and their learning rate was low (Figure 5A), the rewards were relatively less frequent than in WT counterparts, which could add to the demotivation of zQ175 mice during PAL testing. At the same time, the effect of gross locomotor disturbances, another important confound in HD mouse models, on the performance in PAL and FR/PR tasks may probably be excluded: although we tested fairly mature animals, initial locomotor activity (Figure 2), reward collection latencies, and front beam break rates were similar. The rate of rear infrared beam break was lower in Q175Δ*neo* mice during the last FR5 session (Figure 8H), but it was similar to that in WT mice during the subsequent PR sessions (Figure 9I).

In summary, we have expanded the list of known cognitive deficits in the knock-in zQ175 mouse model of HD by showing a drastic impairment of their object location associative memory in the PAL task. Normal performance of this touch screen task is thought to be dependent on the integrity of the hippocampus, dorsal striatum and prefrontal cortex (Owen et al., 1995; Talpos et al., 2009; Delotterie et al., 2015; Kim et al., 2015), i.e., the areas known to be affected by degeneration or biochemical and physiological disturbances in zQ175 mice (Heikkinen et al., 2012; Smith et al., 2014; Rothe et al., 2015; Covey et al., 2016; Peng et al., 2016; Sebastianutto et al., 2017) in correspondence to similar deficits in HD patients. Although the advantage of the highly translational, touch screen-based approach in the case of PAL is weakened by relatively long time needed to complete the test, using younger animals may potentially decrease the overall test duration. In addition, a recently reported more intensive training regimen for PAL task may be applied to shorten the duration of the experiment (Kim et al., 2016). We also demonstrated that pronounced sensorimotor disturbances in Q175 mouse lines can be detected at early touch screen testing stages, e.g., during “Punish incorrect” stage of pretraining for the PAL task or during FR schedule. Therefore, these shorter routines may be utilised for more expedient studies of pharmacological treatments and other strategies aimed at the rescue of HD-related phenotype.

## Author Contributions

MK, TP, LP and RC designed the study. TOP and MK conducted the experiments. TH and JP provided the resources and advised on experimental design. MK and TOP analysed the data. MK wrote the manuscript.

## Conflict of Interest Statement

All research was conceptualised, planned and directed by scientific staff at CHDI and Charles River Discovery. CHDI is a not-for-profit biomedical research organisation exclusively dedicated to discovering and developing therapeutics that slow the progression of Huntington’s disease. Charles River Discovery Services Finland Oy is a contract research organisation that conducted part of the study through a fee-for-service agreement for CHDI Foundation. At the time of the study, LP and RC were employed by CHDI Management, Inc., as advisors to CHDI Foundation, Inc., whereas TOP, TP, TH, JP and MK were employed by Charles River Discovery Services Finland Oy.

## Footnotes

^1^http://www.informatics.jax.org/allele/key/24931
